# Remembering Paolo Sassone-Corsi

**DOI:** 10.1038/s41419-020-02975-z

**Published:** 2020-09-15

**Authors:** Maurizio Bifulco, Alessandro Usiello

**Affiliations:** 1 Department of Molecular Medicine and Medical Biotechnologies, University of Naples “Federico II”, 80131 Naples, Italy; 2 CEINGE Biotecnologie Avanzate, 80145 Naples, Italy; 3 Department of Environmental, Biological and Pharmaceutical Science and Technologies, Università degli Studi della Campania “Luigi Vanvitelli”, 81100 Caserta, Italy

**Keywords:** Medical research, Genetics research

Some men are meant to leave a mark of their passage. Some, more than others, are remembered for their talent, their ability to translate into simple words things that appear distant and impossible to achieve to most of us.

Paolo Sassone-Corsi was one such man. He was one of the great minds of our time who were able to elevate the reputation of Italy in the world. Sadly, Paolo has suddenly passed away. He was a special person, an outstanding colleague, and a first-class scientist. He was able to amaze us with his creativity and boldness. He first worked in Italy, then in France and finally in the USA. He was able to cross the boarders of knowledge in thousands of ways.

He was born in Naples in 1956. From an early age, he had a love for science, particularly astronomy. Together with his brother Emilio, who was a talented astronomer, he founded the Neapolitan Astronomy Club (Gruppo Astrofili Napoletani). Their studies were awarded the Philips Prize for Young Researchers. The two brothers worked at the Paris Meudon Observatory where they studied Saturn. As Paolo once told, “When we were 10 and 11 years old, my brother and I become passionate about astronomy. We were fascinated by the night sky. We weren’t rich, our parents made many sacrifices to buy us a small telescope. I was very lucky because the first thing we saw was Saturn. That was probably the most important moment of my life. It was so amazing. Ever since, for me there was nothing else but nature and science”.

From early studies on the greatest objects in the Universe, Paolo moved to the study of the smallest, namely genes. The fascination for the Universe gave way then to biology and genetics. Paolo once said, “Cicardian biology incorporated both my passions”. However, astronomy remained dear to him. Indeed, his brother Emilio recalled that, also if from different places, they recently watched the comet Neowise together, and were reminded of the emotions they shared in their early observations.

Paolo graduated in biology and specialized in molecular genetics at the University of Naples Federico II. He left Naples when he was 23 years old to pursue a research career abroad. First he moved to Strasbourg and then to the USA where he founded and directed the “Center for Epigenetics and Metabolism” at the University of California, Irvin, where he worked on circadian rhythms and epigenetics.

He was one of the many “sons” of Naples who, thanks to their work, prestige, and personality, were able to spread the Neapolitan culture and mentality. Paolo brought his smile into the temples of science by sharing the sun, the sea, and the Neapolitan joy and creativity with all who knew and loved him. He was always willing to share his knowledge with everyone regardless of academic titles, and always with a smile and simplicity, irrespective if he was speaking to students, researchers or famous scientists.

From the many collaborations and exchanges he had with fellow scientists around the world, it becomes clear that Paolo was widely appreciated and respected. “He was a mentor and an inspiration for all who worked with him”, recalls Nicholas S. Foulkes, his first student in Strasbourg in 1989, “Having worked with him for over 10 years in the early stages of my career, I had the unique opportunity to work alongside and benefit from this truly visionary scientist who always seemed to know ahead of time, what the next major step in his research field would be”.

Paolo was the scientist we all dream of becoming. He sailed the seas of science without fear of any Pillars of Hercules. He accomplished an impressive series of discoveries which, taken together, contributed to our understanding of how genome sequences are decoded and thereby support cellular activity.

The impact of his work is reflected in an incredible number of scientific articles published in the top journals, such as Nature, Science and Cell, and by the many international awards he received throughout his career. His studies and discoveries on transcriptional factors, circadian rhythms, and epigenetics are milestones of biology and will always be a light for new generations of researchers.

His main interest was the study of the rhythms that regulate our biology, thereby avoiding behaviors that may damage our well-being, and also to understand how to improve treatment efficacy.

In his own words: “Life is regulated by cyclic activities aligned to the day and night rhythm. Circadian rhythms, from the Latin circa diem i.e. “around the day”, are involved in virtually every aspect of our behavior and physiology. Circadian rhythms regulate the wake-sleep cycles, metabolism, hormone secretion, immune responses, and behavior, just to mention a few. Consequently, interruption of these rhythms may have physiopathological consequences, including insomnia, depression, diabetes, obesity, early ageing and some forms of cancer”.

Paolo was always hungry for science and knowledge. With the writer Erri De Luca he published a book in 2013 entitled “Ti sembra il caso? Schermaglia fra un narratore e un biologo” (“Do you think it’s by chance? A dispute between a narrator and a scientist”) that showed us Paolo under a different light. Erri and Paolo, both Neapolitan, “We come from the same Mediterranean crossroad. We were linked by birth to Naples, but we moved from there to play our cards on tables far away …” as De Luca recalls. The two become friends thanks to many later exchanges of letters regarding DNA, the circadian clock, the sense of smell, the rhythms that determine the response of the human body and mind to external stimuli and, more generally, to the Universe.

We would like everyone to know, particularly the younger generations, what Paolo gave us, his passion for science, the ability to be moved by a discovery without losing the simplicity and humility. We will always treasure the memory of such a great man and prestigious scientist. We will miss him! Our deepest sympathies go to his wife, Emiliana Borrelli, who is also a talented scientist, and to his family and his lab.

